# *Water Will Be the Coal of the Future*—The Untamed Dream of Jules Verne for a Solar Fuel

**DOI:** 10.3390/molecules21121638

**Published:** 2016-11-29

**Authors:** Vladimir K. Ryabchuk, Vyacheslav N. Kuznetsov, Alexei V. Emeline, Yurii M. Artem’ev, Galina V. Kataeva, Satoshi Horikoshi, Nick Serpone

**Affiliations:** 1 Faculty of Physics, Saint-Petersburg State University, Ulyanovskaya Str. 1, Petergof, Saint-Petersburg 198504, Russia; v.ryabchuk@spbu.ru (V.K.R.); vn.kuznetsov@spbu.ru (V.N.K.); alexei.emeline@spbu.ru (A.V.E.); 2 Laboratory of Photoactive Nanocomposite Materials, Saint-Petersburg State University, Ulyanovskaya Str. 1, Petergof, Saint-Petersburg 198504, Russia; yurii54@yandex.ru; 3 Moscow Engineering Physics Institute, National Research Nuclear University MEPhI, Kashirskoe highway, 31, Moscow 115409, Russia; gvkatayeva@mephi.ru; 4 Department of Materials and Life Sciences, Faculty of Science and Technology, Sophia University, 7-1 Kioicho, Chiyodaku, Tokyo 102-8554, Japan; horikosi@sophia.ac.jp; 5 PhotoGreen Laboratory, Dipartimento di Chimica, Universita di Pavia, Via Taramelli 12, Pavia 27100, Italy

**Keywords:** photosplitting of adsorbed water, solid supports, metal oxides, alkali halides, hydrogen evolution

## Abstract

This article evokes the futuristic visions of two giants, one a writer, *Jules Verne*, who foresaw water as the coal of the future, and the other a scientist, *Giacomo Ciamician*, who foresaw the utilization of solar energy as an energy source with which to drive photochemical and photocatalytic reactions for the betterment of mankind. Specifically, we examine briefly the early work of the 1960s and 1970s on the photosplitting of free water and water adsorbed on solid supports, based mostly on metal oxides, from which both hydrogen and oxygen evolve in the expected stoichiometric ratio of 2 to 1. The two oil crises of the 1970s (1973 and 1979) spurred the interest of researchers from various disciplines (photochemistry, photo-catalysis and photoelectrochemistry) in search of a *Holy Grail* photocatalyst, process, or strategy to achieve efficient water splitting so as to provide an energy source alternative to fossil fuels. Some approaches to the photosplitting of water adsorbed on solid insulators (high bandgap materials; E_bg_ ≥ 5 eV) and semiconductor photocatalysts (metal oxides) are described from which we deduce that metal oxides with bandgap energies around 5 eV (e.g., ZrO_2_) are more promising materials to achieve significant water splitting on the basis of quantum yields than narrower bandgap photocatalysts (e.g., TiO_2_; E_bg_ ≈ 3.0–3.2 eV), which tend to be relatively inactive by comparison. Although proof of concept of the photosplitting of water has been demonstrated repeatedly in the last four decades, much remains to be done to find *the Holy Grail* photocatalyst and/or strategy to achieve significant yields of hydrogen.

## 1. Introduction

Water as a (source) fuel to replace coal was proposed long ago by the well-known French author, Jules Verne, who in his 1874 book *L’Ile Mystérieuse* (The Mysterious Island) [1] stated*…that water will one day be employed as a fuel, that hydrogen and oxygen that constitute it, used singly or together, will furnish an inexhaustible source of heat and light, of an intensity of which coal is not capable. Someday the coal rooms of steamers and the tenders of locomotives will, instead of coal, be stored with these two condensed gases, which will burn in the furnaces with enormous caloric power…I believe, that when the deposits of coal are exhausted, we shall heat and warm ourselves with water… Water will be the coal of the future….* That fuels could be produced from solar energy as the driving force was also an implicit dream of Giacomo Ciamician of the University of Bologna (Italy), known as the father of photochemistry, who in his 1912 lecture at a Conference in New York City stated that… *if in a distant future the supply of coal becomes completely exhausted, civilization will not be checked by that, for life and civilization will continue as long as the sun shines! If our black and nervous civilization, based on coal, shall be followed by a quieter civilization based on the utilization of solar energy that will not be harmful to progress and to human happiness…* [2,3]. These two giants, one a writer and the other a scientist, predicted what we now take for granted, namely that a solar fuel, hydrogen, can be produced from the photodecomposition of water (sometimes also referred to as water splitting).

Back to the future, the *photosplitting of water* was reported for the first time (as early as 1967) by Kotel’nikov and Terenin for water molecules adsorbed on dispersed γ-Al_2_O_3_ particulates [4]; this was later followed by the works of Basov and coworkers on the photodecomposition of water adsorbed on the surface of some oxides, alkali halides and other adsorbents [5,6]. To our knowledge, the work of Kotel’nikov and Terenin [4] described thoroughly the photodecomposition of both water and ammonia adsorbed on dispersed γ-Al_2_O_3_ as well as the photoreduction of oxygen.

The seminal paper that followed by Fujishima and Honda in the journal *Nature* (1972) on the photoassisted electrolysis of water [7] preceded the 1973 oil crisis and stimulated considerable interest in the search for alternative (renewable) sources of energy, and further demonstrated that electron transfer events can occur across a UV-irradiated semiconductor (TiO_2_) photoelectrode/ electrolyte interface. These authors suggested that water can be decomposed by visible light into oxygen and hydrogen without the intervention of an external bias (Reactions (1)–(4)).

TiO_2_ + 2 hν → 2 e^‒^ + 2 h^+^   excitation of TiO_2_ by light (1)

2 h^+^ + H_2_O → ½ O_2_ + 2 H^+^   at the TiO_2_ electrode (2)

2 e^‒^ + 2 H^+^ → H_2_   at the Pt counter-electrode (3)

H_2_O + 2 hν → ½ O_2_ + H_2_   overall reaction (4)

**Figure 1:**
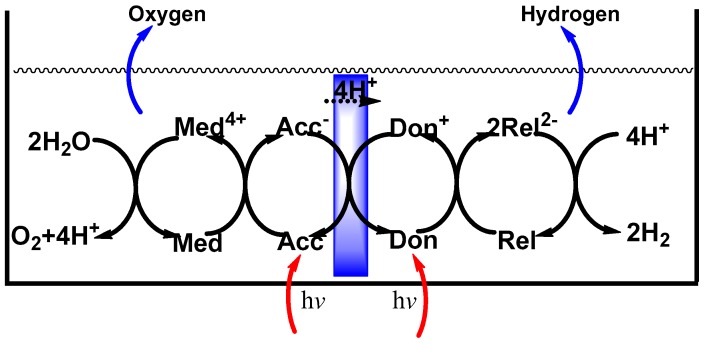
Scheme of the sensitized photosplitting of water through the use of two photochemical reactions working in series: Don = donor dye molecule; Rel = electron relay species; Acc = acceptor dye molecule; Med = electron mediator species. The membrane is permeable to both electrons and H^+^ ions.

The proposal to generate hydrogen through the water splitting process raised the curiosity of several photochemists and photoelectrochemists as this photoassisted process offered an attractive alternative energy resource to replace fossil fuels, a target that could be produced under eco-friendly conditions. There was general consensus in the mid-1970s that production of hydrogen, a suitable energy vector toward establishing a Hydrogen Economy, was a reasonable and environmentally friendly objective to exploit the abundant and inexhaustible sunlight as the energy source that could drive the water splitting process. Along these lines, Balzani and coworkers [8] considered various metallic redox couples (e.g., Ce^4+^/Ce^3+^, Cr^3+^/Cr^2+^ and Eu^3+^/Eu^2+^), binuclear complexes and metal hydrides as possible catalysts in various cycles that could lead to the splitting of water without going through the formation of radical intermediates. The most interesting cycles involved metal hydrido complexes and binuclear complexes in which two metal atoms are bound into a macrocyclic ligand. Bolton later described qualitatively and quantitatively (*Science*, 1978) the thermodynamic and kinetic limitations of the photochemical conversion and storage of solar energy, assessed possible processes with particular attention on producing solar hydrogen from water, and developed a general scheme that might prove useful in this task [9]. A reasonable goal for solar energy storage efficiency in a fuel-generation process was estimated to lie around 10%–13% (probably more [10]). Bolton argued that the most efficient energy storage process was one in which two photosystems operate in series in one-electron redox reactions (Figure 1).

The excitement that prevailed in the decade 1975–1985 to find fuels cleaner than fossil fuels and avoid other oil crises (another one occurred in 1979) by means of the photodecomposition of water failed to materialize in developing catalytic cycles in homogeneous aqueous media to achieve water splitting. Failure to do so led Bard and coworkers to consider formation of hydrogen from water in a heterogeneous aqueous medium using a semiconductor photocatalyst [11,12]; this methodology has come to be known as Heterogeneous Photocatalysis. Within the present context, Bard and Fox [13] sought the *Holy Grail* renewable energy source that could be driven by solar energy and produce a clean and storable fuel, and described it thus: *We want an efficient and long-lived system for splitting water to H_2_ and O_2_ with light in the terrestrial (AM 1.5) solar spectrum at an intensity of one Sun. For a practical system, an energy efficiency of at least 10% appears to be necessary. This means that the H_2_ and O_2_ produced in the system have a fuel value of at least 10% of the solar energy incident on the system.* Subsequently, several articles appeared that emphasized various strategies to achieve evolution of hydrogen from water: (a) by irradiation of water with 185 nm vacuum UV light [14]; (b) photocatalytically by means of various semiconductor photocatalysts (see, e.g., [15]); and (c) photoelectrochemically with various semiconductor photoelectrodes (see, e.g., [16,17,18,19]).

The 2014 article by Protti and coworkers [20] revealed that experimental yields of the solar fuel Hydrogen from the photosplitting of water were disappointingly low despite nearly four decades of intensive research efforts. To understand the possible causes that led to such low yields, we recently identified and discussed several intrinsic and extrinsic factors (e.g., photoexcitation of photo-catalysts, recombination losses of photocarriers, photostability, and back reactions among others) that impact the water splitting reaction, particularly the case of the four-electron process [21]. A large number of studies have focused on titania-based materials to effect water splitting that resulted in low yields of products [22,23,24,25,26,27,28]. Thus, perhaps, we should look elsewhere, for example, though counterintuitive, to wider bandgap (≈5 eV) metal oxides and/or to additional strategies that have yet to evolve. Accordingly, the present article focuses on some of the early efforts to convert light energy into a solar fuel (hydrogen from water) to illustrate some of the important features of the photodecomposition or, better still, of the photosplitting of water that occurred on various wide bandgap metal oxides (E_bg_ >> 3 eV) and others with reference to some selected studies [5,6,29,30,31]. Results from these implicitly argue that the wider bandgap materials deserve a closer look either separately or else incorporated into some novel composite heterostructures.

Commonly used approaches to the photosplitting of water are typically multidisciplinary, involving, as it were, heterogeneous catalysis, photochemistry, photoelectrochemistry and materials science, among others.

## 2. Approaches to the Photosplitting of Water

Studies into the spectroscopic properties and photochemical transformations of simple highly colored molecules (e.g., I_2_, Br_2_, NO_2_ and others) adsorbed on dispersed wide bandgap white solids began in the 1930s (see, e.g., [32] and references therein; [33]) that ultimately led to consider water for its transformation into its constituent hydrogen and oxygen.

Two general mechanisms were proposed for the photosplitting of water pre-adsorbed on a solid adsorbent. The pioneering studies of De Boer [33], De Boer and Houben [34], and Terenin [35] considered the photodissociation of molecules adsorbed on a solid surface as occurring through direct photoexcitation into the red-shifted absorption bands of the molecules. By contrast, Basov and coworkers [6] considered the formation of •OH radicals and surface-bound hydroxyl groups to originate from the primary dissociative adsorption of water on photogenerated surface-hole centers (O_s_^•‒^) following illumination of a metal-oxide (MO) solid (Equations (5) and (6)). This was ascertained from observations that photolysis of adsorbed water in the presence of CO_2_ and CO_2_/CH_4_ mixtures produced formaldehyde (H_2_CO).

M^2+^O_s_^2‒^ + hν → M^+^O_s_^•‒^ (5)

M^+^O_s_^•‒^ + H_2_O → M^+^OH_s_^‒^ + •OH_s_ (6)

Several oxide and alkali halide solids were examined as supports (adsorbents) for the photochemical reactions occurring on their surfaces. Table 1 summarizes the relative activities of some metal oxides, alkali halides and other solids toward the photodecomposition of adsorbed water, with data taken from the work of Basov and coworkers [5,6]. The initial rates of H_2_ evolution with the various solids are compared relative to the rate for BeO (activity set arbitrarily to 100) under irradiation by non-monochromatic light from a 120-W high-pressure Hg lamp (SVD-120).

Measurements for all samples were carried out under standardized conditions (same for all) for water adsorption and subsequent irradiation. Note that the relative activities reported in Table 1 should be taken as approximate as the conditions for a given sample were not optimized because of differences in the number of photons absorbed by each sample. Moreover, the activities of the samples strongly depend on sample preparation (e.g., KBr and HfO_2_ [5]). In nearly all cases, the rates of hydrogen evolution were faster than those for oxygen evolution during water decomposition, as oxygen remained mostly chemisorbed on the solid’s surface; subsequent heating at elevated temperatures released the photolytic chemisorbed oxygen into the gas phase.

**Figure 2:**
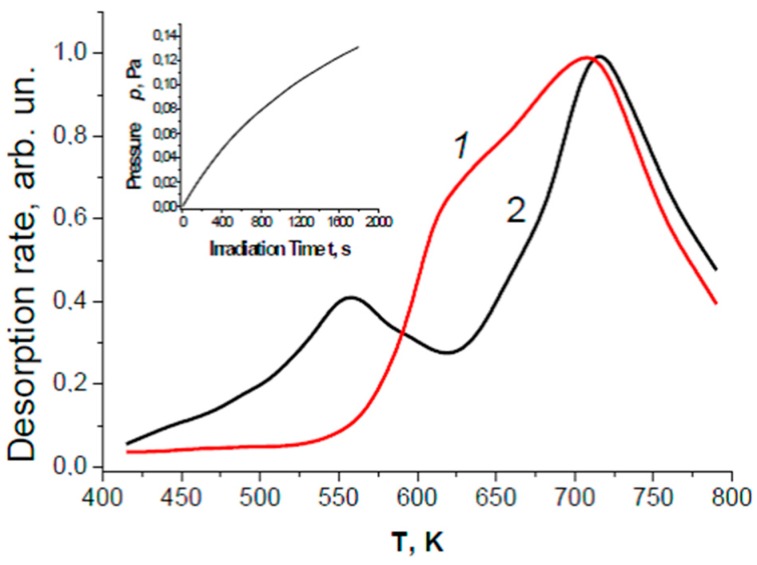
TPD spectra of molecular oxygen for powdered KBr: (1) after photolysis of adsorbed water (curve ***1***); and (2) after photoadsorption of oxygen (curve ***2***). In all cases, irradiation of the samples was performed with a high pressure Hg lamp (DRK-120) whose incident photon irradiance was 5 × 10^14^ photon·cm^‒2^·s^‒1^ at λ ≤ 250 nm; irradiation time for water photolysis was 2000 s; the amount of photolytic and chemisorbed oxygen was nearly 2 × 10^15^ molecules; volume of reactor was ca. 100 cm^3^; front illuminating area of the reaction cell was ~5 cm^2^. Insert shows the kinetics of H_2_ evolution in the gas phase during water photolysis.

Figure 2 displays the thermal desorption spectra (TDS) of oxygen for KBr powders (super fine grade “3–4”, USSR standard, micron scale grain size) after photoadsorption of molecular oxygen and after photolysis of adsorbed water. The spectrum of water photolysis (curve ***1***) partly coincides with the spectrum of photoadsorbed oxygen (curve ***2***) near 700 K. The low temperature band at 550 K for the photoadsorbed oxygen is suppressed completely in the spectrum of the photolysis of adsorbed water probably because of the relatively high activity of weakly bound oxygen in the back reaction of hydrogen oxidation occurring on KBr. This is one reason why molecular oxygen is often not detected in low-activity (photo)catalysts. At elevated temperatures, however, both oxygen and hydrogen evolved in stoichiometric quantities with some increase in the reaction kinetics [6].

One immediate striking feature from the results in Table 1 is that the most active solids are those with the highest bandgap energies. A few additional observations are worth noting from the earlier studies [6,29,30,31] and from the data in Table 1:

(1) Among the active photocatalysts are most of the alkali halides [29], the non-reducible metal oxides (e.g., BeO, MgO, CaO, γ-Al_2_O_3_, and SiO_2_) and the reducible metal oxides (e.g., La_2_O_3_ and ZrO_2_). These oxides differ with regard to dissociative and molecular adsorption of water molecules at regular and defective surface sites [30].
(2) A noticeable activity is shown by the oxides (BeO, γ-Al_2_O_3_ MgO, and SiO_2_) and alkali halides (except KI, RbI, and CsI) with bandgap energies, E_bg_, or energy of the exciton bands for alkali halides [29], greater than 6.2 eV. Accordingly, photoexcitation of such solids by (non-vacuum) UV/visible light into their fundamental absorption bands was not possible.
(3) The red limit of the photosplitting of adsorbed water lies in the rather narrow energy range of 5.4–4.75 eV (230–260 nm) for all the samples examined. The shift of the red limit for the photosplitting of water adsorbed on metal oxides and alkali halides is about 1.0–1.75 eV. A red limit (240 nm or 5.15 eV) within this interval was also reported for the photolysis of water adsorbed on the Pt(111) surface at 85 K [31].
(4) Illumination of samples with adsorbed water evolves H_2_ into the gas phase, while oxygen tends to remain adsorbed at the surface. Photolytic oxygen can be detected in some cases in the gas phase on heating the samples after irradiation (Table 1). At times, formation of oxygen in the reaction manifested itself via formation of secondary products (e.g., CO) through interaction of oxygen with some surface organic contaminants. In the case of a few catalysts (BeO and ZrO_2_ are good examples [5,6]) oxygen began to evolve into the gas phase in the expected stoichiometric ratio for water splitting (at a definite time delay) after prolonged illumination at ambient temperature.
(5) The rate of photodecomposition of water on metal oxides can usually be enhanced by moderate heating of the samples in the presence of adsorbed water. This infers some selective activity of chemisorbed water species.
(6) In the case of BeO, for which experiments were carried out using a water vapor flow photoreactor, molecular hydrogen and molecular oxygen evolved in the gas phase in stoichiometric amounts at elevated temperature (T = 200 °C) under UV illumination for a few hours without decrease in activity [6]. A noticeable decrease of the reaction rate occurred, however, under prolonged illumination at ambient temperature for this oxide and other solids.

**Figure 3:**
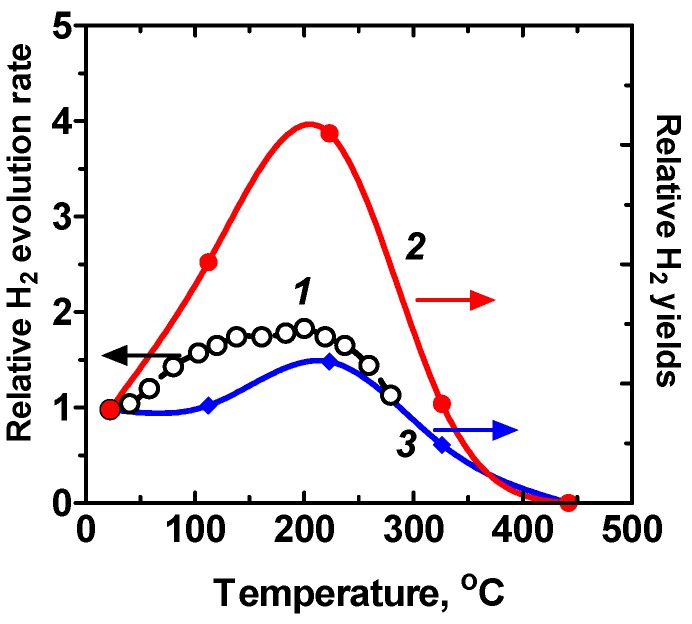
Temperature dependences of the relative rate of hydrogen evolution (curve ***1***) during the photolysis of adsorbed water on BeO (water vapor pressure = 2 × 10^2^ Pa); the temperature dependence of oxygen evolution was similar to that for hydrogen; the gases H_2_ and O_2_ evolved in amounts close to stoichiometric [5]. The relative hydrogen yields in the radiolysis of water on ZrO_2_ at 2.8 MeV electrons (curve ***2***) and at 5.0 MeV He^2+^ ions (curve ***3***) are also displayed. Curves ***2*** and ***3*** were adapted with permission from Roth et al. *J. Phys. Chem. C* **2012**, *116*, 1719 [36]. Copyright 2012 American Chemical Society.

The temperature dependences of the relative rates of hydrogen evolution occurring during the photolysis of water adsorbed on BeO [6] and, for comparison, the relative yields of hydrogen during the radiolysis of water adsorbed on ZrO_2_ are also reported in Figure 3. The temperature dependence of oxygen evolution (not shown) was similar to that for hydrogen; the gases evolved in amounts close to stoichiometric [6].

To the best of our knowledge, there are no complete sets of data that describe a reaction pathway for the photolysis of adsorbed water in metal oxides/adsorbed water systems. For instance, it is not known which stage(s) of adsorbed water dissociation on BeO determines the increase of the reaction rate with temperature from ambient to 200 °C (Figure 3) [6,36]. Nonetheless, a few typical features of the photosplitting of adsorbed water are worth noting and are discussed next.

### 2.1. Adsorbed Active Water Species Subsequent to UV-Induced Dissociation

The existence of a potential energy barrier for the chemisorption process and a relatively deep potential well can be inferred in the formation of active water species during the photosplitting of adsorbed water for a large number of solid adsorbents, such as the alkali halides [29] and the metal oxides [5,6]. Bullet (5) above is a rather common feature in the photolysis of adsorbed water. Indeed, the initial rate of photolysis of adsorbed water occurring 15 min after the first contact of water vapor with the BeO surface, subsequently heated in the presence of oxygen at 800 °C, is six- to six-fold lower than the maximal rate of photolysis owing to prolonged (a few days) contact of BeO with water vapor at ambient temperature [5,6]. Similarly, the initial rate of water photolysis after a 30-min period of heating the water vapor samples at 100 °C on a series of potassium halides (KF, KCl, KBr and KI) was, respectively, 1.4-, 4.0-, 1.5-, and 3.3-fold greater than those before heating. Note that the samples were placed under vacuum after cooling to ambient temperature; no physisorbed molecules remained at the surface. In the Kotel’nikov and Terenin studies [4], alumina samples were heated to 600 °C in a water vapor atmosphere for 6 h to enhance the efficiency of the reaction before determination of the quantum yields. No hydrogen evolution was detected on irradiation of strongly hydroxylated alumina samples in the absence of adsorbed water. The same was confirmed for a hydroxylated BeO surface in similar control experiments [10].

Aging the metal-oxide and alkali-halide samples in water vapor, or their pre-heating to enhance the dissociation of adsorbed water, is not related to the activation of adsorption of water but to the formation of surface hydroxyl groups which, in turn, favor the formation of active adsorbed water species. The latter was also emphasized in the earlier studies of Basov and coworkers [5,6]. Water decomposition taking place on BeO [37] was monitored by mass-spectrometric and IR spectroscopic methods; adsorbed water species were characterized by the infrared absorption bands at 3686 cm^‒1^ and at 3282 cm^‒1^. These IR bands were assigned to the valence vibrations of water molecules with one of the OH groups hydrogen-bonded to a surface oxygen atom. Photolytically active water species desorbed from the BeO surface upon heating the samples to 450–470 K [37], in accord with the studies of Kuznetsov and Lisachenko [10] who observed no evolution of H_2_ upon removal of adsorbed water by sample heating and out-pumping at T > 473 K. Thus, the decrease of the reaction rate at T > 200 C (curve ***1***, Figure 3) is likely the result of desorption of photolyzed active water species. Using the simple Readhead model [38], the activation energy of desorption of active water species is about 1.0–1.6 eV (frequency factor within 10^7^–10^13^ s^‒1^); water desorption displayed a maximal first-order at temperatures between 440 and 475 K. The activation energies of desorption of two active adsorbed water species, also estimated by the Readhead model, were 1.4 eV and 1.7 eV (pre-exponential frequency factor, 1 × 10^13^ s^‒1^), respectively, for the He^2+^-ion induced (5.0 MeV) and electron-induced (E = 2.8 MeV) radiolytic dissociation of water on ZrO_2_ powders [36].

In general, both photostimulated photolysis of adsorbed water and high-energy stimulated dissociation of water occur during the primary excitation of zirconia; the surface hydroxyl groups are unaffected by the action of the high-energy radiation and by the action of UV light (Figure 3). Both pathways of stimulated dissociation of adsorbed water have many common features [39,40]. For instance, IR spectroscopic data led Roth and coworkers [36] to consider water radiolysis as occurring with neutral adsorbed water molecules bound to the terminal Zr−OH (3760 cm^−1^) and bridged Zr−OH−Zr (3660 cm^−1^) surface hydroxyl groups. Both He^2+^-ion and electron impact radiolytic dissociation of adsorbed water displayed maximal rates at 200 °C that were subsequently reduced to noise level at 400 °C. No significant hydrogen evolution was detected from the destruction of surface OH groups under high-energy irradiation of ZrO_2_. This temperature dependence was also observed in the photosplitting of adsorbed water occurring on BeO [6] (curve ***1***, Figure 3).

Results indicate that the rather strongly bound adsorbed water molecules undergo photolysis on various alkali halides and metal oxides. Hydroxylation of the solid’s surface favors the light-induced dissociation of water and so infers that surface hydroxyls take part in water-surface coupling; yet these hydroxyls are relatively UV-resistant.

## 3. Photoexcitation of Adsorbed Water

Two approaches to excite adsorbed molecules that lead to their subsequent decay can be distinguished: absorption of photons by the adsorbed molecules versus absorption of light by the solid photocatalyst. Strictly speaking, absorption of light by adsorbed *molecules* implies weak interactions between the solid’s surface and the molecules. In the general case, *an adsorbed molecule* should be treated as a surface defect of the solid particle (herein the substrate) characterized by specific electronic states and a corresponding absorption spectrum that is associated more with light absorption by the solid’s defect than to light absorption by the molecule. In turn, photoexcitation of adsorbed molecules via the substrate refers to *intrinsic* or *extrinsic* absorption of light by the solid particle and to subsequent excitation of the adsorbed molecules (herein the species). The latter process may occur either via energy transfer by mobile quasi-particles (e.g., electron–hole pairs or excitons), or by some resonance mechanism.

### 3.1. Direct Photolysis of Adsorbed Water Molecules

Red-shifted absorption bands of adsorbed molecules (e.g., Br_2_, NO_2_, and NH_3_) were considered in the early studies ([32] and references therein; [33]) as the primary photoinduced process toward their dissociation on photoinactive surfaces of silica, alumina, and alkali and alkali earth halides. A discussion on the direct photoexcitation of adsorbed water (Table 1) requires we first consider, albeit briefly, the photolysis of free (non-adsorbed) H_2_O molecules.

Free H_2_O molecules are dissociated by absorption of photons with energies 6.5 eV < hν < 8.8 eV yielding H• and •OH radicals in their ground electronic states (Equation (7)):

H_2_O + hν → H_2_O* → H• + •OH (7)

where H_2_O* denotes water in the unstable dissociative electronic state ^1^B_1_. The quantum yield of Reaction (7) is close to unity [14,41,42]. Following a complex series of events, secondary reactions of H• and •OH radicals lead to the ultimate formation of dihydrogen and dioxygen [14]. These radicals are also the intermediates in photocatalytic water splitting in solid photocatalyst/liquid water systems that are formed through reductive (H^+^ + e^‒^ → H•) and oxidative (OH^‒^ + h^+^ → •OH) stages, respectively. At least two photons are needed for dihydrogen formation in Reactions (3) and (7) provided back reactions of the intermediates are excluded. The maximal theoretical quantum yield of H_2_ formation is 0.5, while for O_2_ the expected quantum yield is 0.25.

Another spin-allowed path for the photolysis of free water involves direct photoexcitation of water to its higher excited states, with particular formation of molecular hydrogen in the ground electronic state and excited oxygen atom (^1^D) at photon energies E(hν) ≥ 7.0 eV. In the latter case (Equation (8)), only one photon is needed to produce dihydrogen from water molecules and two photons for dioxygen. The quantum yield of reaction (8) is less than 0.01 [41,42] and requires high energy photons. Consequently, Reaction (8) is less promising than the process represented by reaction (7), an analog of photoexcitation of adsorbed water.

H_2_O + hν → H_2_O* → H_2_ + O (^1^D) (8)

Basov and coworkers [5,6] considered the photosplitting of water as occurring via photon absorption by adsorbed water that experiences a red shift of its absorption spectra as a result of perturbation effects on the solid’s surface. They also monitored the evolution of hydrogen and oxygen (Table 1) from (water) ice under UV illumination; the observed red limit was at λ = 230–250 nm (5.4–5.0 eV). In this regard, Bachler and Gartner [43] showed that the external electric field needed to cause a red shift of the first absorption band of water (Equation (7)) to ~300 nm is ca. 3 × 10^8^ V·cm^‒1^ upon adsorption of water onto a solid surface; this is an extremely high value for an internal crystal field. Nonetheless, the bathochromic shift of the water absorption bands under such a high field conditions can play a definite role in the photolysis.

### 3.2. Photolysis of Adsorbed Water Molecules via Photoexcitation of the Solid Photocatalyst

Results from the photolysis of adsorbed water show that the main pathway for excitation of water toward its decomposition is energy transfer from the light-activated solid photocatalyst. In this regard, Table 2 reports the red limits of the photosplitting of adsorbed water, together with the photoreduction of oxygen and photooxidation of hydrogen (sometimes referred to as photo-adsorption of oxygen and hydrogen, respectively) for a set of semiconductor/insulator photo-catalysts. It should be emphasized that the photoreduction of O_2_ and photooxidation of H_2_ on metal-oxide solids are stoichiometric reactions of oxygen and hydrogen with surface-active centers with trapped photoelectrons and photoholes, respectively. These reactions occur because of the photogeneration of electrons and holes and thus serve, to some extent, as models of the oxidative and reductive half-reactions of photocatalytic reactions.

Table 2 also reveals: (i) red shifts of 1.6–3.4 eV in the photolysis of adsorbed water compared to free H_2_O molecules (6.5 eV); (ii) red limits of both the photolysis of adsorbed water and of the oxidative and reductive half-reactions occurring on irradiating into absorption bands far from the fundamental absorption threshold of the solid photocatalysts; and (iii) the spectral regions of oxidative and reductive half-reactions are either the same (ZrO_2_ as an example) or else overlap (MgO as an example) [44]. The latter suggests that both electrons and holes are generated by photo-excitation of the solids on irradiation in the overlapping absorption bands of electron- and hole-type defects that model, to some extent, band-to-band excitation of the photocatalysts. In the case of photon absorption followed by formation of so-called bound excitons and their subsequent decay to free charge carriers and/or centers with trapped carriers, the red limits of the final process of creation of surface-active centers will be the same—see for example the studies by Sterrer and coworkers [45], Diwald et al. [46], and Volodin [47]. Both electrons and holes can be generated by photoexcitation of wide bandgap solids such as Al_2_O_3_ [48] via the photoconversion of F-centers, subsequent to absorption of two low-energy photons by *intrinsic* defects which yield free electrons and holes and causes the restoration of these defects to their initial concentration. This approach was used by Kuznetsov and Lisachenko [49] to explain the spectral dependence of the photoadsorption (i.e., photoreduction) of oxygen on magnesia and alumina (see below).

Accordingly, the pair of interconvertible defects F^+^ ↔ F (anion vacancies with one and two trapped electrons, respectively) may be considered as *self-sensitizing* entities for the generation of electron/hole pairs under sub-bandgap photoexcitation of the solid photocatalyst.

### 3.3. Quantum Yields of the Photosplitting of Adsorbed Water and the Photoadsorption of O_2_ and H_2_ on Metal Oxides

Precise experimental quantum yields of the photolysis of adsorbed water have been reported for a few metal oxides that include Al_2_O_3_ [6], HfO_2_ [50], and BeO [10]. The quantum yield of the photolysis of adsorbed water on α-Al_2_O_3_ at a photon energy E_hν_ ≈ 6.0 eV (i.e., at 210 nm) is Φ_210(H2)_ = 0.27 ± 0.08, which represents the highest quantum yield reported so far. For water dissociation occurring on HfO_2_, the quantum yield Φ_254(H2)_ lies in the range 3–7 × 10^‒5^ [50], while for BeO the quantum yield Φ_206(H2)_ is 2.2 ± 0.2 × 10^‒2^ [10]. Poston and coworkers reported *apparent quantum yields* at 185 nm (6.7 eV) and at 254 nm (4.9 eV) for the photolytic process occurring on monoclinic *m-*ZrO_2_ and cubic *c*-ZrO_2_ nanopowders [39]; the activity of *m*-ZrO_2_ was about tenfold greater for the photolysis of water adsorbed on monoclinic *m*-ZrO_2_ than for *c*-ZrO_2_. These authors [39] associated this difference to the enhanced structural imperfections of stabilized *c*-ZrO_2_ that, in turn, led to an effective recombination of the photocarriers and the excitons, as well as to a decrease of the photocatalytic activity of this zirconia polymorph. However, the activities at λ = 185 nm of *m*-ZrO_2_ and *c*-ZrO_2_ samples were about an order of magnitude greater than the activities at λ = 254 nm. The *true quantum yields* of water photolysis on *m-*ZrO_2_ at 185 nm was Φ_185(H2)_ ≈ 0.10‒0.15 and did not exceed 0.01 at 254 nm.

Contrary to the data presented in Table 1, *m*-ZrO_2_ appears more active under photoexcitation at 185 nm than BeO at 206 nm on the basis of the quantum yields of water decomposition. Discrepancies in the relative activities of the metal oxides, based on either the experimental quantum yields at a fixed wavelength and/or the integral rates of H_2_ evolution (Table 1), are likely due: (i) to differences on how photocatalyst activity is defined physically; (ii) to dependencies of photocatalyst activity on the state of the adsorbed water (see Bullet (5), Section 2); (iii) to the dependence of the activity on such factors as particle size, surface morphology, and nature of the defects among others; (iv) to the dependence of the quantum yield of decomposition of the adsorbed water on the surface concentration of adsorbate species when the photodriven reaction occurs via absorption of photons by the solid; and (v) on different spectral dependencies of the quantum yields of different photocatalysts.

**Figure 4:**
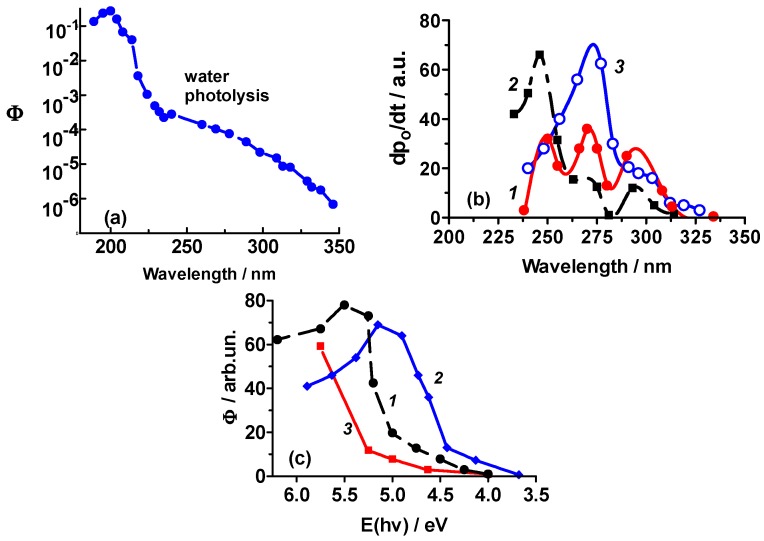
(**a**) Spectral dependences of the quantum yields of the photolysis of water on α-Al_2_O_3_ particles. The surface of the solid adsorbent had previously been hydrated by heating the alumina particles in a water vapor atmosphere at 250 °C for 6 h—note the logarithmic plot of Φ; (**b**) Spectral dependences of the initial rate of the: (1) photodecomposition of water; (2) photoreduction of oxygen; and (3) photodecomposition of ammonia on γ-Al_2_O_3_ (from the data reported by Kotel’nikov and Terenin [4]); (**c**) Spectral dependencies of the quantum yields of the photoreduction (photoadsorption) of oxygen on: (1) γ-Al_2_O_3_; (2) MgO; and (3) BeO [49]. Note: In Ref. [49], the spectral dependencies of Φ were presented in arbitrary units but are nonetheless proportional to the true quantum yields.

The spectral dependence of the quantum yield for the photosplitting of adsorbed water for the H_2_O/α-Al_2_O_3_ system measured in a black-body-like reactor [6,51] is illustrated in Figure 4a (note the logarithmic scale of Φ). The maximal quantum yield is seen at 200 nm and decreases thereafter at longer wavelengths to 350 nm. This is not unusual as the spectral dependencies of the quantum yields of photoreduction of oxygen and photooxidation of hydrogen typically overlap in a rather wide spectral range below the fundamental absorption edge of wide bandgap photocatalysts. For instance, the corresponding red limits of overlapping spectral dependencies of the quantum yields are 3.2–3.3 eV for ZrO_2_ (E_bg_ = 5.4 eV), MgO (E_bg_ = 8.7 eV), and Sc_2_O_3_ (E_bg_ = 6.0 eV) [51].

Figure 4b illustrates the spectral dependence of the initial rate of the photodecomposition of water (curve ***1***) consists of three well-resolved maxima at 250 nm (4.95 eV), 270 nm (4.6 eV), and 290 nm (4.3 eV). These spectral features are better resolved and are different vis-à-vis the features displayed by the true quantum yield (compare curve ***1*** in Figure 4b with Figure 4a) [52]. The spectral dependencies of the quantum yields of the photoreduction (photoadsorption) of oxygen on Al_2_O_3_, MgO, and BeO reported by Kuznetsov and Lisachenko [49] are displayed in Figure 4c.

Typically, the maxima (and minima) in the spectral dependencies of quantum yields points to the existence of a photocatalytically inactive background absorption and/or to an overlap of the photoactive absorption bands related to different light-absorbing centers [52]. Thus, one should not expect a similarity in the shapes of curve ***2*** (Figure 4b) and curve ***1*** (Figure 4c) for the photo-adsorption of oxygen on alumina. The poorly resolved features for the photodecomposition of ammonia (curve ***3***) compared to those of curve ***1*** for the photodecomposition of water on alumina (Figure 4b) are also worth noting. In addition, the positions of the two maxima and the shoulder in the spectral dependence of the photoreduction of oxygen (curve ***3***, Figure 4b) coincide with those for the photodecomposition of water (curve ***1***). The latter bears witness of the photosplitting of adsorbed water via absorption of photons by alumina in the spectral region λ ≥ 250 nm at which the quantum yield of water photolysis is rather low: <10^‒3^ for α-Al_2_O_3_ (Figure 4a).

## 4. Mechanistic Considerations of the Photosplitting of Adsorbed Water

The simultaneous generation of electrons and holes under illumination of a solid support (photocatalyst) in the *extrinsic* absorption region may be associated with: (i) the photoionization of defects (color centers) in the spectral region corresponding to the overlap of absorption bands of various types of defects (either electron or hole); (ii)intrinsic and extrinsic surface defects; and (iii) some specific mechanism of the transformation of F-centers that also lead to the generation of electron/hole pairs. In this regard, the spectral region of the photosplitting of adsorbed water on Al_2_O_3_ (Figure 4a,b) and MgO (Table 2) allows considerations of all three possibilities. Related to this, Kuznetsov and Lisachenko [49] noted that electron/hole generation in the *extrinsic* absorption region for Al_2_O_3_ and MgO occurs by conversion of F-centers since the positions of the F and F^+^ absorption bands (5.5 eV and 4.8 eV for Al_2_O_3_ and near 5.0 eV for MgO) favor the process (curve ***1***, Figure 4c).

In general, the photodecomposition of adsorbed water by photoexcitation into the *extrinsic* absorption bands follows a mechanistic course similar to the generation of electron/hole pairs proposed by Garibov and coworkers [53] for the γ-ray-induced production of hydrogen from water adsorbed on Al_2_O_3_ (Equations (9)‒(11)).

H_2_O_ads_ + h^+^ → H_2_O^+^_ads_ (9)

H_2_O^+^_ads_ + e^‒^ → H_2_O*(_ads_) → H• + •OH (10)

H• + •H → H_2_ (11)

The dissociative adsorption of water molecules (before illumination) taking place following adsorption onto a solid’s surface through homolytic HO–H bond breaking leads to the formation of two •OH species as a result of the coupling of H• atoms with surface oxygens [30]; different reactions of the adsorbed species with the photocarriers ultimately yields dihydrogen. In the case of multilayer water adsorption, formation of H^+^ and OH^−^ adsorbed species also occurs because of the stabilization of these ions by the surrounding water molecules. In the latter case, the path to the photosplitting of water is similar to what occurs in a solid photocatalyst/liquid water system, rather than to *direct* photolysis of adsorbed water.

The reaction scheme expressed by Equations (9)–(11) is consistent with the absence of multilayers of adsorbed water. Adsorbed water molecules could be neutral and could be taken as specific surface recombination centers that become electronically excited (similar to recombination luminescent centers) after trapping a second carrier (herein a photogenerated electron). Formation of the excited state of adsorbed water molecules (corresponding to an excited state of free molecules) does not follow the usual optical selection rules, so that an alternative reaction pathway may be indicated (Equation (12)):

H_2_O^+^_ads_ + e^‒^ → H_2_O*(_ads_) → H_2_ + O (^3^P) (12)

**Figure 5:**
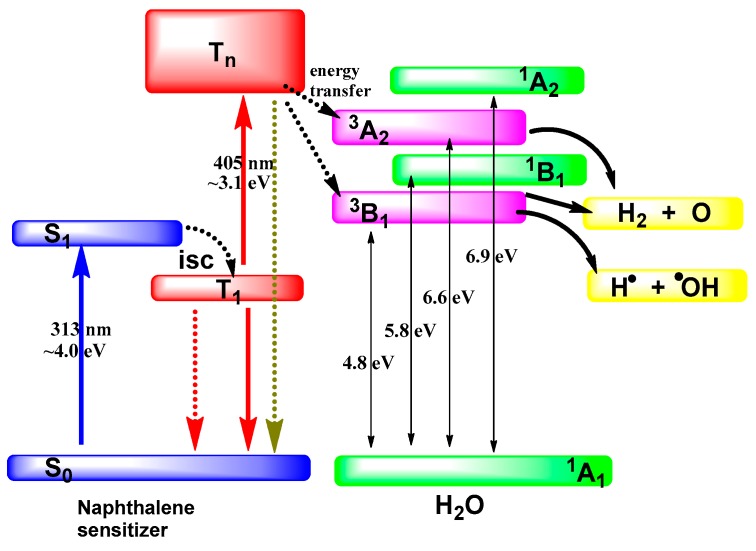
Scheme illustrating the excitation of the naphthalene photosensitizer and triplet-triplet energy transfer from the photosensitizer to excited states of water leading to its photosplitting into H• and •OH radicals, and to an oxygen atom and dihydrogen. Note: The energies for water represent the energy minima of the respective excited states according to Claydon and coworkers [54].

In this case the excited state(s) of water molecules H_2_O*(_ads_) corresponds to the triplet dissociative states ^3^B_1_ and ^3^A_2_ of free water molecules (Figure 5) perturbed by the adsorption process with decay channels leading to the formation of dihydrogen and an oxygen atom in its ground electronic state ^3^P (energy ~5.0 eV) [54]. The lowest energy pathway for water dissociation is optically spin-forbidden since the ground state of water molecules is the singlet ^1^A_1_ state.

The dissociation of adsorbed water via Reaction (12) needs only one photon or one electron/hole pair (excluding different losses) to generate dihydrogen (two photons to form dioxygen) and thus could reflect the high quantum yield. Hence, it is tempting to suggest that Reaction (12) is what likely takes place on excitation of alumina at a photon energy close to 6 eV (ca. 200 nm; Figure 4a). However, to the extent that the observed quantum yield is quite substantive (Φ ≈ 0.3), direct excitation of adsorbed water via a reaction similar to that described by Equation (7) cannot be precluded since a moderate red shift from 6.6 to 6.2 eV, caused by surface perturbation, would be needed in this case. Note that the photosplitting of adsorbed water can occur via different pathways on excitation of the catalyst in different spectral regions.

One may now query whether it is possible to populate the dissociative electronic state of adsorbed water molecules. Germane to this, Korotkov and coworkers [55] contended that direct decomposition of adsorbed water on SiO_2_ in the presence of the photosensitizer naphthalene occurs via population of the water’s lowest excited triplet states ^3^B_1_ and ^3^A_2_ by triplet-triplet energy transfer (Figure 5) from excited naphthalene upon simultaneous irradiation with two photons, one at λ_1_ = 313 nm (ca. 4.0 eV) and the other at λ_2_ = 405 nm (ca. 3.1 eV). Only evolution of dihydrogen from adsorbed water was detected; the corresponding quantum yield was significantly small: Φ_(H2)_ = 7 ± 2 × 10^‒4^ [55].

Notwithstanding the above, however, that photosplitting of adsorbed water takes place through direct light absorption by the adsorbed molecules at the primary stage of the reaction is not unequivocal. The problem is that photoexcitation of the majority of dispersed wide bandgap solids in the spectral range where they are transparent can also generate photoelectrons and photoholes. The efficiency of carrier generation could be significant enough to drive the surface photoreactions (e.g., photoredox reactions of molecules) by photons absorbed in the *extrinsic* absorption band of the solids because of the presence of various crystallite imperfections (e.g., *intrinsic* and *extrinsic* point defects in the bulk and at the surface of the solid particles). Consequently, the point made earlier (Bullet (2) in Section 2) that photoexcitation of the solid into the fundamental absorption band for the photoreaction to occur may not be valid.

The relative activities of various metal oxides in the photosplitting of adsorbed water presented in Table 1 suggest that the increased activity with increase in the actinic photon energy up to, or close to, the energy of the lowest excited states of free water may be rather common (the system H_2_O/γ-Al_2_O_3_ illustrated in Figure 4a is an example). Thus, one of the primary steps of the dissociation of adsorbed water is the decay of its electronic excited state. This assumption finds evidence in the study of Petrik and coworkers [40] on the efficiency of the γ-radiolysis of adsorbed water. It is relevant to recall that excitation of wide bandgap solids by γ-rays is similar to UV (band-to-band) photoexcitation since, after the formation of charge carriers and relaxation of their momentum and energy during the γ-excitation event, the properties of the free carriers—electrons near the bottom of CB and holes near the top of VB—are the same as the carriers generated by UV photoexcitation.

**Figure 6:**
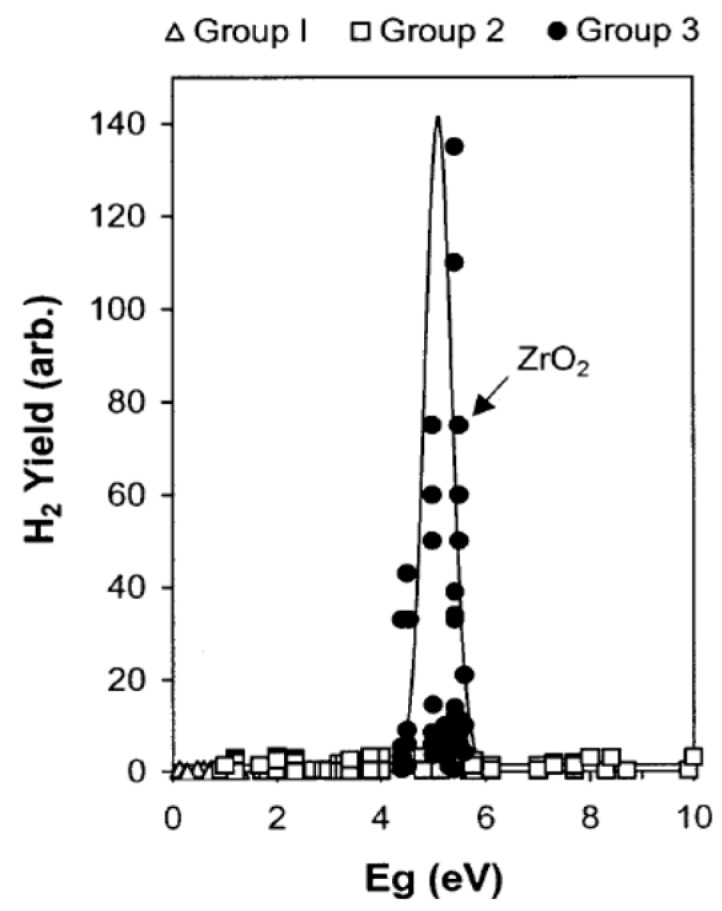
Hydrogen chemical radiation yield versus bandgap of various oxides (E_bg_): Group 1 represents inhibitor metal oxides (e.g., MnO_2_, Co_3_O_4_, CuO, and Fe_2_O_3_), Group 2 represents indifferent metal oxides to γ-radiolysis (e.g., MgO, CaO, SrO, BaO, ZnO, CdO, Cu_2_O, NiO, Cr_2_O_3_, Al_2_O_3_, CeO_2_, SiO_2_, TiO_2_, Nb_2_O_5_ and WO_3_), and Group 3 represents metal-oxide promoters (Ga_2_O_3_, Y_2_O_3_, La_2_O_3_, Nd_2_O_3_, Sm_2_O_3_, Eu_2_O_3_, Gd_2_O_3_, Yb_2_O_3_, Er_2_O_3_, HfO_2_, and ZrO_2_) for the γ radiolysis of adsorbed water. For more details, see Ref. [31]. Reprinted with permission from Petrik et al. *J. Phys. Chem. B* **2001**, *105*, 5935 [40]. Copyright 2001 the American Chemical Society.

Zirconia displays maximal activity for some adsorbed water species with moderate adsorption energies [40]. This is also a characteristic feature of the photolysis of adsorbed water. The decomposition of water adsorbed on ZrO_2_ takes place through energy transfer from the excitons (e°) to the adsorbed molecules at some crucial stage in the process (Equation (13)); i.e., from a coupled electron/hole pair (e°) with energy a few eV less than the bandgap energy of the solid. For the series of metal oxides examined by Petrik et al. [40], a resonant curve emerged from a plot of radiolytic hydrogen yields versus bandgap energy of various metal oxides (Figure 6).

H_2_O + e° → (H_2_O)* → H• + •OH (13)

It is noteworthy that the metal oxides that define the band in Figure 6 are those whose band-gap energies E_bg_ lie close to 5 eV; these are the oxides Ga_2_O_3_, Y_2_O_3_, La_2_O_3_, Nd_2_O_3_, Sm_2_O_3_, Eu_2_O_3_, Gd_2_O_3_, Yb_2_O_3_, Er_2_O_3_, HfO_2_, and ZrO_2_ that make up Group 3 in Figure 6, and represent metal-oxide promoters in the γ-radiolysis of adsorbed water. Group 1 comprises the metal oxides MnO_2_, Co_3_O_4_, CuO, and Fe_2_O_3_, which are inhibitors of the radiolysis of adsorbed water, whereas Group 2 metal oxides comprises MgO, CaO, SrO, BaO, ZnO, CdO, Cu_2_O, NiO, Cr_2_O_3_, Al_2_O_3_, CeO_2_, SiO_2_, TiO_2_, Nb_2_O_5_ and WO_3_, all of which are inconsequential to the radiolytic process.

The existence of an effective channel for the photosplitting of adsorbed water through the lowest electronic excited states ^1^B_1_, ^3^B_1_ and ^3^A_2_ [54,56,57,58,59,60] still cannot be precluded, however. The excited state(s) of adsorbed water molecules (H_2_O* in Equation (13)), treated as an excited state of a surface *extrinsic* defect, can be populated not only via energy transfer from the exciton (e°, Equation (13)), but equally well by direct radiative excitation as a result of photon absorption by or energy transfer to the adsorbed molecules, or via the emitted energy from the trapping of electrons and holes followed by their recombination. That is, the excited state of a defect (adsorbed molecule) populated by different ways may be the same. Regardless of the mechanism of the decomposition of adsorbed water, however, the reported maximal quantum yields tended to be relatively high (Φ = 0.1–0.3).

**Figure 7:**
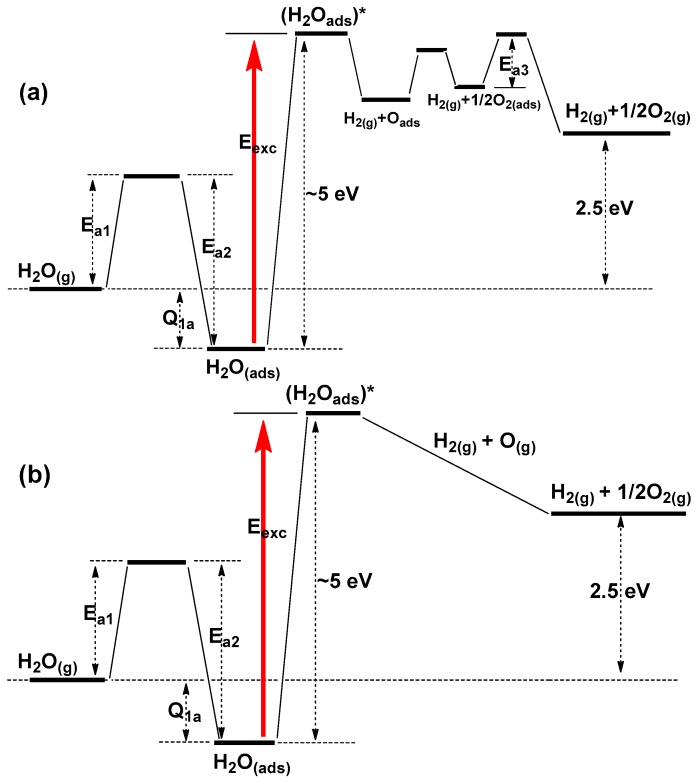
Qualitative schematic illustration of two possible reaction pathways (**a**) and (**b**) for a one-photon induced photolysis of adsorbed water in the presence of a suitable photocatalyst: (**a**) case where photolytic oxygen forms as adsorbed species with subsequent recombination to chemisorbed molecular oxygen and thermally activated desorption; (**b**) case where recombination of the primary water decomposition product occurs in the gas phase without chemisorption.

It is important to emphasize that: (i) the most effective photosplitting of adsorbed water occurs at photon energies around 5 eV, both close to the energy of the lowest electronic excited states of water and to the energy of dissociation of free water into H_2_ and O (^2^P) in their ground electronic states; and (ii) the decomposition of water molecules needs but only one photon (or one exciton or one electron/hole pair generated by an absorbed photon) to be effective to attain the maximal theoretical quantum yield. Figure 7a,b illustrates two generalized reaction pathways of some of the possible mechanistic stages of a one-photon induced photosplitting of adsorbed water.

The activation energy of desorption (E_a2_) of active adsorbed water species (with regard to photosplitting) lies in the range 1.0–1.6 eV for BeO (Section 2.1). For ZrO_2_ powders, E_a2_ was ascertained directly from the thermal desorption spectra as 1.4 eV and 1.7 eV, respectively, from the He^2+^-ion and electron impact radiolytic (E = 2.8 MeV) dissociation of water on this metal oxide [36]. Accordingly, the heat of adsorption of active adsorbed species (Q_ads_, Figure 7a,b) can be represented by the equation Q_ads_ = (E_a2_ − E_a1_) ≤ 1.0–1.7 eV.

The energy of excitation of adsorbed water species (for an effective photolysis with quantum yields greater than 0.1), E_exc_, in Figure 7a,b is ~5 eV, an energy close to both the energy of the lowest excited states of free water molecules and to the energy of water dissociation into H_2_ and O [54,56,57,58,59,60]. Excitation of adsorbed water molecules likely occurs through energy transfer from the solid catalyst via electron/hole recombination or via excitonic mechanisms. In this case, excitation of adsorbed water molecules in the lowest energy triplet states are possible in contrast to direct photo-excitation by photons since the corresponding electronic transitions are spin-forbidden by the Wigner rule. The subsequent decay of excited adsorbed water into H_2_ in the gas phase (H_2(g)_) and an adsorbed oxygen atom (O_ads_) is illustrated in Figure 7a. At the beginning of illumination, the reaction of adsorbed oxygen atoms with residual organic contaminants on the surface typically yields CO and CO_2_ (Table 1; Section 2, Bullet (4)). Exothermic recombination of adsorbed oxygen atoms leads to formation of adsorbed dioxygen species (Figure 7a). The formation of free dioxygen in the gas phase occurs at elevated temperatures (E_3a_). The latter corresponds to results for many known systems in that the stoichiometric evolution of H_2_ and O_2_ takes place at elevated temperatures. For a few systems, the stoichiometric evolution of hydrogen and oxygen was also found to occur at ambient temperature ***after*** prolonged illumination (Section 2, Bullet (4)). Recombination of primary products from the excited water molecules (e.g., oxygen atoms) can occur either in the physisorbed state (Figure 7a) or in the gaseous state (Figure 7b).

We have outlined above one of the most effective pathway for the photodissociation of adsorbed water. Other pathways that require more photons per reaction cycle will obviously be less effective. Accordingly, wide bandgap solids may have a considerable advantage over narrower bandgap solids if the process were to occur via a one-photon pathway. The problem to overcome now is the *UV limit* of the reaction.

## 5. Final Remarks on Photosplitting of Water and Future Outlook

The strategies used to achieve photosplitting of water rest on the usage of solid photocatalysts as photon harvesters, and on their use to transfer the energy of absorbed photons to surface-adsorbed water molecules via photogenerated charge carriers. Loss of photocarriers through recombination within the solid photocatalyst lattice and at the surface is a serious barrier, a sort of *bottleneck*, which impacts the efficiency of the photocatalytic water splitting process.

Wide bandgap solids have demonstrated relatively high activity in the photolysis of adsorbed water, with the most popular photocatalyst TiO_2_ being relatively inactive by comparison, as are other relatively narrower bandgap photocatalysts (Table 1). The rather low activity of titania in relation to the photodecomposition of adsorbed water in comparison with the photoelectro-chemical decomposition of water was recognized some time ago by Schrauzer and Guth [61], Kawai and Sakata [62] and by Smart and coworkers [63]. It is not unlikely, therefore, that this relatively low activity of titania impeded somewhat the progress in photocatalytic water splitting as a large number of studies focused on this photocatalyst in the past. In contrast, wider bandgap metal oxides appear more promising photocatalysts for the photosplitting of adsorbed water as witnessed by the results in Table 1, and may yet provide interesting opportunities toward a practical water splitting process in the not too distant future. In this regard, the use of ZrO_2_ (E_bg_ = 5.4 eV) obtained by a precipitation method to accomplish water splitting was reported nearly a decade ago by Reddy et al. [64] and more recently by Poston and coworkers [39].

Domen and coworkers have examined the systems RhCrO_x_/LaMg_1/3_Ta_2/3_O_2_N and Mo-doped BiVO_4_, embedded together in a Au layer as potential hydrogen evolution photocatalysts and oxygen evolution photocatalysts, respectively, in the form of photocatalyst sheets coated with a protective layer of either anatase TiO_2_ or ZrO_2_ to effect the visible-light driven water splitting process [65]. The TiO_2_-coated sheet was somewhat ineffective because of the low H_2_ evolution activity of RhCrO_x_/LaMg_1/3_Ta_2/3_O_2_N. By contrast, when the surface of LaMg_1/3_Ta_2/3_O_2_N was coated with a ZrO_2_ protective layer the H_2_ evolution activity was enhanced more than threefold in aqueous methanolic media, and the activity of water splitting was nearly doubled under optimized conditions relative to the TiO_2_-covered sheet; however, excessive loading of ZrO_2_ apparently lowered the activity because it inhibited charge transfer between LaMg_1/3_Ta_2/3_O_2_N and the Au layer [65].

An important impediment toward significant product yields (and thus quantum yields) from the photolysis of adsorbed water is associated with back reactions, as the final products from the photosplitting of water (H_2_ and O_2_ molecules) can react with each other under illumination. The photooxidation of H_2_ can lead to a reaction between hydrogen and photoreduced (photoadsorbed) oxygen species (e.g., O_2_^‒•^, O^‒•^), which are intermediates in the water splitting process. The photooxidation of hydrogen was clearly demonstrated for BeO [10], for KBr and other alkali halides [29], and for ZrO_2_ [66], i.e., for catalysts that display relatively high activity in the photosplitting of adsorbed water (Table 1). Back reactions and secondary reactions of primary intermediate radical products of water dissociation (Equations (7), (8) and (13)) may also be very effective. Germane to this, Maeda and coworkers [67] examined a solar-driven Z-scheme approach to effect water splitting employing the Pt/BaZrO_3_−BaTaO_2_N couple as the H_2_ evolution photocatalyst and the IO_3_^−^/I^−^ redox mediator in combination with PtO_x_/WO_3_ and TiO_2_ rutile as two O_2_ evolution photocatalysts. Both systems produced stoichiometric quantities of H_2_ and O_2_ under simulated sunlight irradiation at conversion efficiencies of 0.0067% for the (Pt/BaZrO_3_−BaTaO_2_N + Pt/WO_3_) system and 0.014% for the (Pt/BaZrO_3_−BaTaO_2_N + TiO_2_ rutile) system. A most significant observation was that H_2_ and O_2_ generated from the water splitting process recombined (i.e., back reaction), a reaction that is thermodynamically favored occurring on the metallic Pt nanoparticles in the composite Pt/BaZrO_3_–BaTaO_2_N system during the process. The occurrence of this undesirable reaction during photo-catalytic water splitting decreased the detected rates of evolution of H_2_ and O_2_. Clearly, this back reaction must be suppressed if water splitting by Z-schemes is to be efficient; no H_2_−O_2_ recombination occurred on PtOx/WO_3_ and TiO_2_ rutile [67].

The use of wide bandgap solids with E_bg_ >> 3 eV as effective photocatalysts may necessitate additional thoughts be given to modify these materials in some unusual manner so as to enhance their effective field toward greater absorption of sunlight at wavelengths well beyond 290 nm. A few approaches that have been considered to achieve this and that might also further the activity of wide bandgap photocatalysts in the visible spectral region are: (a) by the so-called *narrowing of the bandgap* whereby the photocatalyst’s lattice is cation- or anion-doped or co-doped [68]; (b) by dye photosensitization [69]; (c) by surface plasmon resonance excitation via noble metals [70]; and lastly (d) by the up-conversion luminescence technique [71]. Recalling that the *magic red limit* of the photolysis of adsorbed water occurs at energies in the range 5.4–4.75 eV, close to both the lowest electronic energy levels of water molecules and to the energy of water dissociation established for a representative number of oxides and halides (Table 2), sensitization of modified wide bandgap photocatalysts by two-photon photoexcitation through a Z-scheme approach seems a promising tactic to perform water splitting with visible-light-active wide bandgap photocatalysts.

Of the few listed methods to photosensitize wide bandgap solids, the up-conversion luminescence method should provide a golden opportunity for future studies. To the best of our knowledge, the UV-up-conversion luminescence technique from photon energy of ~5 eV has yet to be established. Another possibility may be the usage of a modified Z-scheme approach whereby the system is composed of a core consisting of two narrow bandgap semiconductors that can be activated with low energy photons and a shell consisting of a wide bandgap semiconductor as proposed by Serpone and Emeline [72]. Another promising way to achieve photosensitization of wide bandgap metal oxides is to design and examine suitable composites that take advantage of the surface enhanced Plasmon resonance (SEPR) slow-photon-effect on photonic crystals [73] coupled to hybrid Z-sheme heterostructures with wide bandgap semiconductor (or insulator) components similar to the Ag_2_S–Ag–TiO_2_ hybrid structures described by Li and coworkers [74].

At the present stage of research, the *Holy Grail* photocatalyst, process, or strategy has yet to be discovered [21].

## Figures and Tables

**Table 1:** Relative activities of dispersed solids used in the photodecomposition of adsorbed water with respect to the activity of BeO set arbitrarily at 100. Taken in part from Ref. [5].

Solid	Bandgaps (eV)	Relative Activity (Arbitrary Units)	Products Detected in the Gas Phase from the Photosplitting of H_2_O	
BeO	10.5	100	H_2_	O_2_
Be(OH)_2_	-	30	H_2_	O_2_
γ-Al_2_O_3_	9.5	30	H_2_	O_2_
ZrO_2_	5.0	10	H_2_	O_2_
La_2_O_3_	5.5	9	H_2_	O_2_
ThO_2_	5.7	8	H_2_	O_2_
Na_2_(AlF_6_)	-	7	H_2_	O_2_
H_3_BO_4_	-	6	H_2_	O_2_
HfO_2_	5.6	6	H_2_	O_2_
SrO	5.8	6	H_2_	O_2_
SiO_2_	8.2	3	H_2_	O_2_
Ho_2_O_3_	5.4	1	H_2_	O_2_
Gd_2_O_3_	5.3	<1	H_2_	O_2_
Sc_2_O_3_	6.3	<1	H_2_	O_2_
MgO	7.6	<1	H_2_	O_2_
TiO_2_	3.2	<<1	H_2_	-
ZnO	3.4	<<1	H_2_	-
GeO_2_	5.6	<<1	H_2_	-
Yb_2_O_3_	3.0	<<1	H_2_	-
Dy_2_O_3_	4.9	<<1	H_2_	-
Nb_2_O_5_	(3.5)	<<1	H_2_	-
Zeolite NaX	-	<<1	H_2_	O_2_
KBr	7.5	7.0	H_2_	-
KCl	8.7	0.4	H_2_	-
NaCl	8.5	0.5	H_2_	-
H_2_O (“snow”)	-	1.0	H_2_	O_2_

Taking the activity of KBr in arbitrary units as being 7.0, and comparing the units in terms of the results reported for KBr (insert in Figure 2) and from the fact that the same grade of KBr (super fine grade) was used and exposed to similar pretreatment procedures and similar irradiation conditions used both in Ref. [5] and in Ref. [29], the relative activity 7.0 for KBr corresponds to the amount of evolved hydrogen, 4 × 10^15^ molecules, for an illumination period of 2000 s (insert in Figure 2). Note also that the mean value of the rate of water photolysis is approximately 2 × 10^12^ molecules·s^‒1^ (or 3 × 10^‒6^ μmol·s^‒1^ or ~10^‒2^ μmol·h^‒1^). The irradiated area of the KBr sample was ca. 5 cm^2^, so that if we take the depth of UV light penetration into the powdered sample to be near 10 μm, then the rate of H_2_ evolution estimated for the KBr adsorbent (density, 2.75 g·cm^‒2^) is 0.7 μmol·g^‒1^·h^‒1^ at an incident irradiance of ~5 × 10^14^ photons·cm^‒2^·s^‒1^ in the spectral region of KBr photoactivity (λ ≤ 250 nm). Thus, the highest activity assigned as 100 to BeO reflects ~10 μmol·g^‒1^·h^‒1^ of hydrogen under illumination with the 120-W high pressure Hg lamp.

**Table 2:** Bandgap energies and red limits of the photoreduction of oxygen, photooxidation of hydrogen and the photosplitting of adsorbed water. Taken in part from Refs. [32,44].

Solids	E_bg_ (eV)	Red Limits of Photoreactions		
		Photoreduction of O_2_ (eV)	Photooxidation of H_2_ (eV)	Photosplitting of H_2_O (eV)
TiO_2_	3.2	2.2	~2.2	~3.1
ZnO	3.2 (3.4) *	1.7	-	-
MgO	8.7 (7.6) *	4.0	2.7	4.9
γ-Al_2_O_3_	9.5	3.75	~4.0	3.75
MgAlO_4_	9.0	4.75	4.75	-
MgAlO_4_ (Cr)	9.0	3.0	3.0	-
ZrO_2_	5.4 (5.0) *	3.1	3.1	-
LiF	13.6	3.9	4.9	4.9
NaF	11.6	3.9	4.9	4.9
KBr	7.5	3.7, 1.6	-	4.9
SiO_2_	>10 (8.2) *	4.95	-	4.95
SiO_2_ (V)	>10	-	3.7	-
SiO_2_ naphthalene	>10	-	-	3.9

* Ref. [44].
